# Corrigendum: Modeling crowdsourcing as collective problem solving

**DOI:** 10.1038/srep33105

**Published:** 2016-09-12

**Authors:** Andrea Guazzini, Daniele Vilone, Camillo Donati, Annalisa Nardi, Zoran Levnajić

Scientific Reports 5: Article number: 1655710.1038/srep16557; published online: 11 10 2015; updated: 09 12 2016

This Article contains errors. In the ‘Model’ section,

“Capacity is simply the number of iterations in which at least one collectivist has solved the task, regardless of *R*. It models the collective knowledge of a group, i.e., the group’s capacity to solve increasingly challenging tasks. It is defined for each group, regardless of its iteration-dependent divisions into collectivists and individualists. Initially we set for all groups Σ_*j*_ = 0. We update Σ_*j*_ → Σ_*j*_ + 1 each time at least one player playing as a collectivist solves the task (and regardless of how many individualists solve it)”.

should read:

“Capacity is simply the number of iterations in which one collectivist has solved the task, regardless of *R*. It models the collective knowledge of a group, i.e., the group’s capacity to solve increasingly challenging tasks. It is defined for each group, regardless of its iteration-dependent divisions into collectivists and individualists. Initially we set for all groups Σ_*j*_ = 0. We update Σ_*j*_ → Σ_*j*_ + 1 each time one player playing as a collectivist solves the task”.

Additionally in this section,

“The group’s capacity increases by one Σ_*j*_ → Σ_*j*_ + 1. Fitness updates are as follows: …”

should read:

“The group’s capacity increases by *S* Σ_*j*_ → Σ_*j*_ + *S*. Fitness updates are as follows: … ”

In the ‘Results’ section,

“… where, by definition, 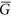 = 11/5 is the mean value of the integer random number G_*i*_ ∈ {1, 2, …, 10}”.

should read:

“…where, by definition, 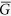 = 11/2 is the mean value of the integer random number G_*i*_ ∈ {1, 2, …, 10}”.

Additionally in this section,

“This clearly implies that *δ* increases monotonically with *R*, going from 0 to 11/5. This means that, with increasing *R* cooperative groups have to reach a higher capacity to thrive, but this is balanced by the higher ease to solve the tasks: the combined effect is that, given the group size, the final cooperation level does not depend strongly on *R*, as shown in Fig. 1. On the other hand, increasing *R* enhances the global fitness, because it is easier to solve the tasks and, for a cooperative group, reach a higher capacity with respect to the others, as depicted in equation (5). This behavior is confirmed in Fig. 3, where an increase for increasing *R* is observable in the average agent fitness. The narrow tilt change for *R* ≃ 0.5 can be understood considering that when *R* becomes smaller than R* = 0.5, also δ gets smaller than ¬¬ δ* ≃ 0.5, that is, there is practically no need to reach a higher capacity for cooperators to thrive, lowering the global fitness”.

should read:

“This clearly implies that *δ* increases monotonically with *R*, going from 0 to 11/2. This means that, with increasing *R* cooperative groups have to reach a higher capacity to thrive, but this is balanced by the higher ease to solve the tasks: the combined effect is that, given the group size, the final cooperation level does not depend strongly on *R*, as shown in Fig. 1. On the other hand, increasing *R* enhances the global fitness, because it is easier to solve the tasks and, for a cooperative group, reach a higher capacity with respect to the others, as depicted in equation (5). This behavior is confirmed in Fig. 3, where an increase for increasing *R* is observable in the average agent fitness. The narrow tilt change for *R* ≃ 0.5 can be understood considering that when *R* becomes smaller than R* = 0.5, also δ gets much smaller than ¬¬ δ* ≃ 0.5, that is, there is practically no need to reach a higher capacity for cooperators to thrive, lowering the global fitness”.

